# When the Vessels Whisper: The Power of Multimodality Imaging in Takayasu Arteritis

**DOI:** 10.1016/j.jaccas.2025.104446

**Published:** 2025-06-25

**Authors:** Jan M. Brendel, Patrick Krumm

**Affiliations:** a Cardiovascular Imaging Research Center (CIRC), Department of Radiology, Massachusetts General Hospital, Harvard Medical School, Boston, Massachusetts, USA; b Department of Radiology, Diagnostic and Interventional Radiology, University of Tuebingen, Tuebingen, Germany

**Keywords:** heart failure, inflammatory large-vessel vasculitis, magnetic resonance angiography, vascular disease, vascular imaging

**Figure:**
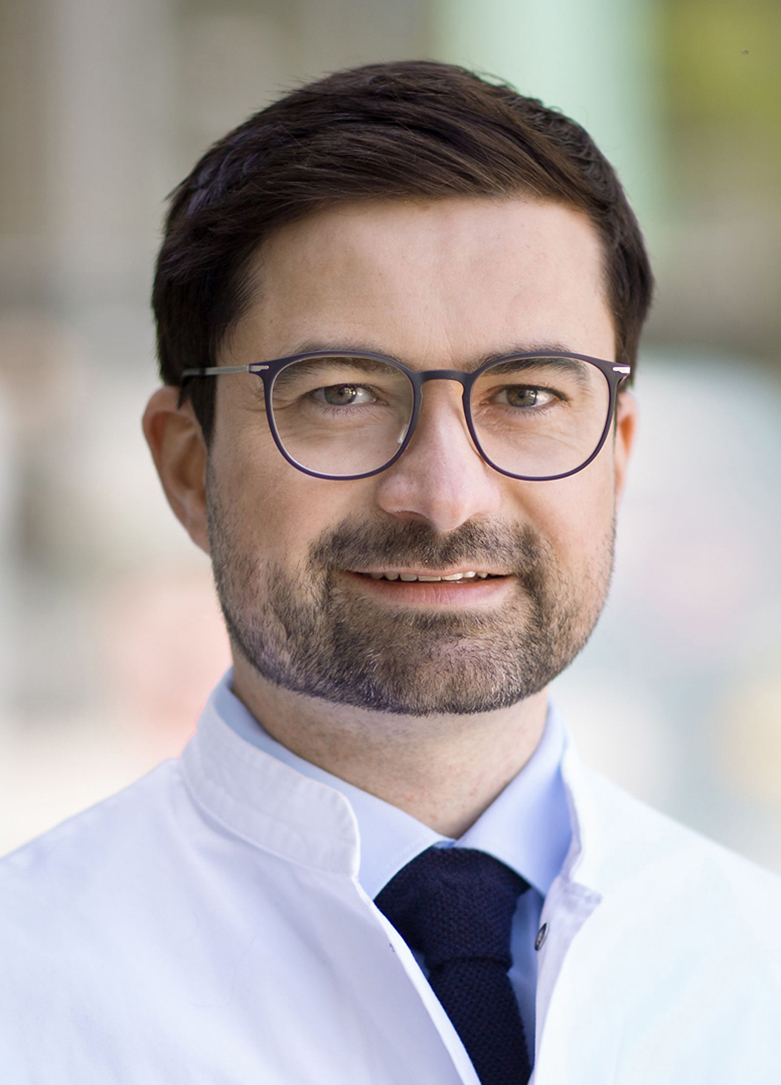

Takayasu arteritis (TA) remains a diagnostic conundrum in cardiovascular medicine. It is a chronic inflammatory vasculitis affecting the large arteries, particularly the aorta and its major branches. Predominantly seen in young women, TA is characterized by granulomatous inflammation, leading to vessel wall thickening, stenosis, occlusion, or aneurysm formation. Clinical presentation is highly variable, contributing to diagnostic delay and complexity in management.

In this issue of *JACC: Case Reports*, Tao et al^1^ report the case of a 16-year-old Asian female patient presenting with heart failure with reduced ejection fraction, persistent headaches, hypertension, and a history of patent foramen ovale closure.

## When Diagnostic Challenges Converge

The clinical narrative was complex; the early symptom of severe headache was initially attributed to the patent foramen ovale, and subsequent viral pneumonia and myocarditis further obscured the underlying vascular inflammation. This case is emblematic of the diagnostic challenges inherent to TA, where nonspecific systemic signs and concomitant cardiac abnormalities may mislead even experienced clinicians. The authors deserve commendation for their methodical approach in re-evaluating the patient's symptoms, which ultimately led to the consideration of TA.

## The Role of Multimodality Imaging

The diagnostic turnaround in this case was significantly driven by the use of advanced imaging modalities. Magnetic resonance angiography (MRA) or computed tomography angiography should be performed to evaluate arterial narrowing, occlusion, and inflammatory vessel wall involvement.^2^ For repeat studies, particularly in younger patients, MRA is usually preferred because it lacks radiation. Color Doppler ultrasound can complement MRA/computed tomography angiography by evaluating superficial vessels, particularly the carotid and subclavian arteries, but may not sufficiently visualize deeper arteries.^3^ In the present case, in combination with cardiac magnetic resonance, which revealed features suggestive of myocarditis, these techniques provided a comprehensive picture of both vascular and myocardial involvement. Further, positron emission tomography, in combination with computed tomography or magnetic resonance, is increasingly used to aid diagnosis by detecting active arterial inflammation.^4^

In brief, appropriate comprehensive imaging can shorten the time to diagnosis and treatment, thereby reducing the risk of irreversible vascular damage and associated complications.^5^ The case presented clearly illustrates these principles and serves as a call to action for clinicians to incorporate these imaging modalities into their diagnostic work-up for patients with unexplained cardiovascular and systemic findings.

## Potential Adverse Outcomes of Delayed Diagnosis

The natural history of untreated or late diagnosed TA is associated with significant adverse outcomes.^6^ In the absence of timely intervention, progressive arterial inflammation can lead to critical complications such as severe renovascular hypertension, myocardial ischemia, and ultimately irreversible heart failure. Cerebrovascular events also loom large, as persistent vascular inflammation can precipitate cerebral ischemia—a scenario that may manifest as persistent headache or even stroke. In this case, the patient's early symptoms of headache and episodic hypertension were harbingers of the underlying vasculitic process. Had these clues been fully appreciated in the preoperative period, the clinical course may have been favorably altered. This case report serves as a reminder that the integration of clinical vigilance with advanced imaging is not just an academic exercise—it is a critical determinant of patient prognosis.

## Therapeutic Implications

Once the diagnosis of TA was established, the initiation of immunosuppressive therapy, including intravenous methylprednisolone and oral methotrexate, led to a notable improvement in myocardial function. The partial recovery of left ventricular ejection fraction from 29% to 41% within 1 month of therapy is encouraging. This confirms the advances in medical therapy over the past few decades, demonstrating that timely treatment can halt disease progression and partially reverse cardiac damage, even in advanced cases.^7^ Nonetheless, invasive procedures remain an effective option to treat critical vessel lesions,^8^ and lifelong surveillance is essential for long-term outcomes.

## Conclusions

In summary, the case presented illustrates how careful clinical evaluation combined with advanced imaging can resolve complex diagnostic challenges in TA. As clinicians and diagnosticians, we are reminded of the importance of maintaining a broad differential diagnosis in young patients with unexplained heart failure and persistent systemic symptoms, and that the path from symptom onset to diagnosis can be filled with pitfalls. Ultimately, the integration of multidisciplinary clinical expertise and comprehensive imaging remains the approach of choice in addressing complex vasculitides, such as TA.

## Funding Support and Author Disclosures

Dr Brendel is funded by the 10.13039/501100001659Deutsche Forschungsgemeinschaft (DFG, German Research Foundation) (#540505270). Dr Krumm is a member of the Speakers Bureau for Siemens Healthineers and 10.13039/501100000801Bayer Healthcare.
